# Prebreeding studies of spring bread wheat lines, carrying Т1BS.1BL-1S#F7L-1BL translocation with Lr51 gene

**DOI:** 10.18699/vjgb-26-87

**Published:** 2026-10

**Authors:** S.N. Sibikeev, A.E. Druzhin, Z.E. Fitileva, I.V. Borisov

**Affiliations:** Federal Center of Agriculture Research of the South-East Region, Saratov, Russia; Federal Center of Agriculture Research of the South-East Region, Saratov, Russia; Federal Center of Agriculture Research of the South-East Region, Saratov, Russia; Federal Center of Agriculture Research of the South-East Region, Saratov, Russia Institute of Biochemistry and Physiology of Plants and Microorganisms – Subdivision of Saratov Scientific Centre of the Russian Academy of Sciences, Saratov, Russia

**Keywords:** spring bread wheat, lines-analogous of cultivar Saratovskaya 68, T1BS.1BL-1S#F7L-1BL, resistance to leaf rust, influence for grain productivity and bread making quality, яровая мягкая пшеница, линии-аналоги сорта Саратовская 68, Т1BS.1BL-1S#F7L-1BL транслокация, устойчивость к листовой ржавчине, влияние на продуктивность и качество зерна

## Abstract

One of the sources of bread wheat resistance to the Puccinia triticina is the Lr51 gene in the T1BS.1BL-1S#F7L-1BL translocation, from Aegilops speltoides Tausch. However, it is little used in the practical breeding of Triticum aestivum L. The main reason is the fear of the negative impact of translocation on important agronomic features. This paper presents the results of pre-breeding studies of lines-analogues of spring bread wheat cultivar Saratovskaya 68 – L6 and L7, carrying T1BS.1BL-1S#F7L-1BL translocation, compared with the recipient – Saratovskaya 68 and the standard – Saratovskaya 76. The presence of the Lr51 gene was confirmed by PCR analysis with markers S30-13L and AGA7-759R. Lines L6 and L7 are resistant to saratov populations of P. triticina. The lines-analogues are cytologically stable. The presence of translocation in L6 and L7 did not significantly influence for plant height and lodging resistance. In lines-analogues, the duration of the germination-earing period is at the level of recipient and standard cultivars. For grain productivity, lines L6 and L7 do not significantly differ from Saratovskaya 68. However, lines L6 and L7 surpassed Saratovskaya 76 in 2024, but there were no differences for 2023–2025 period. Analysis of the structure of the elements productivity of the main ear for average 2023–2025 period showed that at the lines L6 and L7 no differences with the cultivar-recipient for all traits. But comparisons with Saratovskaya 76, show significant differences in favor of lines with translocation in terms of ear density, while L7 also had significant differences for grain weight from the ear and number grains in spikelet.The presence of the T1BS.1BL-1S#F7L-1BL translocation in L6 and L7 did not significantly affect the protein content, as well as the indicators of flour and bread, except for the volume and porosity of bread. Lines L6 and L7 were inferior to Saratovskaya 76 in terms of elasticity, the ratio of elasticity to extensibility (P/L), and the volume of bread, but surpassed the strength of flour (W). In general, in terms of cytological stability and the entire range of breeding-valuable indicators, the effect of T1BS.1BL-1S#F7L-1BL translocation in the genotype of the Saratovskaya 68 is assessed as neutral, and lines L6 and L7 have breeding significance for leaf rust resistance.

## Abstract (ru)

Одним из эффективных генов устойчивости к возбудителю листовой ржавчины (Puccinia triticina) у мягкой пшеницы (T. aestivum L.) является ген Lr51, интродуцированный в геном мягкой пшеницы от Aegilops speltoides Tausch в составе транслокации T1BS.1BL-1S#F7L-1BL. Однако этот ген редко используют в практической селекции из-за опасения негативного влияния чужеродной транслокации на агрономические важные признаки. В данной работе представлены результаты пребридинговых исследований яровых линий мягкой пшеницы Л6 и Л7, несущих T1BS.1BL-1S#F7L-1BL транслокацию, по сравнению с сортом-реципиентом Саратовская 68 и сортом-стандартом Саратовская 76 по комплексу селекционно значимых признаков. Наличие гена Lr51 в генотипе линий Л6 и Л7 подтверждено ПЦР-анализом с использованием маркеров S30- 13L и AGA7-759R. Фитопатологическая оценка линий Л6 и Л7 в условиях искусственного и естественного инфекционного фона к локальной популяции листовой ржавчины, распространенной в Саратовской области, показала их высокий уровень устойчивости в сравнении с сортом-реципиентом. По оценке цитологической стабильности линий, мейотический индекс составляет 98.2 и 99.7 у линий Л6 и Л7 соответственно. Наличие транслокации у Л6 и Л7 не влияло значимо на высоту растений и устойчивость к полеганию. У линий-аналогов продолжительность периода всходы–колошение оказалась на уровне сорта-реципиента и сорта-стандарта. По продуктивности зерна линии Л6 и Л7 также не отличались достоверно от сортов Саратовская 68 и Саратовская 76, по данным полевых испытаний за период 2023–2025 гг. Анализ структуры продуктивности главного колоса у линий Л6 и Л7 выявил отсутствие различий с сортом-реципиентом по всем показателям. По сравнению с сортом Саратовская 76 были достоверные различия в пользу линий-аналогов по плотности колоса, а у Л7 также по весу зерна с колоса и озерненности колоска. Наличие транслокации Т1BS.1BL-1S#F7L- 1BL у Л6 и Л7 незначимо повлияло на содержание белка, а также на качество муки и хлеба. Однако обе линии уступали сорту-реципиенту по таким признакам, как объем и пористость хлеба. Линии Л6 и Л7 уступали сорту Саратовская 76 по упругости, отношению упругости к растяжимости, объему хлеба, но превзошли по силе муки. В целом, с учетом цитологической стабильности и комплексу селекционно ценных показателей, влияние Т1BS.1BL-1S#F7L-1BL транслокации в генотипе сорта Саратовская 68 оценивается как нейтральное, а линии Л6 и Л7 имеют селекционную значимость по устойчивости к листовой ржавчине.

## Introduction

Bread wheat (Triticum aestivum L. em. Thell) is one of the main food crops. Wheat accounts for over 27 % of the total global grain production. Intensive breeding for increased productivity of this crop has led to a significant reduction of its gene pool in terms of resistance genes to diseases and pests. The susceptibility of bread wheat varieties to diseases leads to huge losses in grain yield and a decrease in its quality. For example, average losses from leaf rust in Russia and foreign countries are 4–6 %, and under epiphytotic conditions of this pathogen, grain yield losses can reach 50–70 % (Dymchenko et al., 1990). In recent decades, an increase in the harmfulness of the disease spectrum of bread wheat has been noted for the Lower and Middle Volga regions of Russia. A severe stem rust epiphytotic was observed in 2016, a severe leaf rust and septoria leaf spot epiphytotic in 2017, a severe tan spot epiphytotic in 2019, a local but severe stem rust epiphytotic in 2020 and simultaneous moderate epiphytotics of stripe and leaf rust, powdery mildew, and tan spot in 2025. There is constant pressure on bread wheat plants from fungal diseases. Thus, the importance of increasing the yield of cultivated plants through resistance to biotic factors, which include a large group of phytopathogens, is sharply increasing. To avoid economically significant losses from bread wheat diseases, including rust species, constant scientifically based breeding for resistance to pathogens is necessary. This work should be based on knowledge of the pathogen’s biology, its virulence, the resistance genetics of cultivated crops varieties, and a sufficient number and diversity of resistance genes to pathogens, i. e., it should be anticipatory (McIntosh, Brown, 1997). The most difficult task is to expand the genetic diversity of resistance genes to pathogens. For example, studies by O.A. Baranova (2020) on a set of 32 new bread wheat cultivars entered into the State Register of Selection Achievements of the Russian Federation for 2017–2018 showed that only 11 cultivars have high effectiveness against the stem rust pathogen. Of these, seven cultivars are protected by one gene Sr31, three cultivars by a combination of Sr31+Sr57 and one cultivar Sr31+Sr28. Studies by E.I. Gultyaeva et al. (2025) on a set of 28 winter and 16 spring bread wheat cultivars approved for use in 2024 showed that seven winter and five spring bread wheat cultivars are resistant to the leaf rust pathogen, i. e., 25 and 37 %. However, only one winter bread wheat cultivar is resistant to the stripe rust pathogen.

Currently, effective resistance to pathogens in the majority of wheat cultivars is due to the presence of resistance genes introgressed from wild and cultivated relatives of bread wheat, rather than their own resistance genes. The species Aegilops speltoides Tausch 2n = 14, genome S (Schneider et al., 2008) should be particularly highlighted. Twelve identified genes transferred from this species are known, including six genes for resistance to leaf rust (Lr28, Lr35, Lr36, Lr47, Lr51, Lr66), three to stem rust (Sr32, Sr39, Sr47) and three to powdery mildew (Pm12, Pm32, Pm53) (grainsgenes.org/GG3/wgc, accessed February 11, 2026). I.G. Adonina et al. (2012) identified the T5BS.5BL-5SL translocation from Ae. speltoides, which carries an effective gene for leaf rust resistance, designated as LrASP5. Among the identified genes, the gene Lr51 (laboratory designation LrF7), obtained from crossing the bread wheat cultivar Neepawa with Ae. speltoides source F, should be noted, and then six backcrosses were made to the cultivar Neepawa and a highly resistant line F7 to the leaf rust pathogen was obtained (Dvorak, 1977). In this line, a translocation from chromosome 1S Ae. speltoides to chromosome 1BL of the cultivar Neepawa – T1BS.1BL-1S#F7L-1BL (Dvorak, Knott, 1980) was localized. Later, in one derivative line from F7 – F-7-3, an intercalary translocation from 1S to 1BL ranging from 14 to 32 cM was established, including the loci XAga7 and Xmwg710 (Helguera et al., 2005). The gene Lr51 for resistance to the leaf rust pathogen is semidominant and highly effective both in Russia (Gultyaeva et al., 2024, 2025), and abroad, particularly in the USA (Helguera et al., 2005). Despite the effectiveness of resistance, this gene is rarely used in commercial bread wheat cultivars. It is known from the sources available to us that the Lr51 gene has been identified in ten Egyptian bread wheat cultivars (Ashraf et al., 2015). Among Russian cultivars, this gene has not been identified (Gultyaeva et al., 2025). At the National Center of Grain named after P.P. Lukyanenko (FSBSO “NCG P.P. Lukyanenko”), a series of lines with combinations of Lr51 with Lr genes have been obtained: Lr10, Lr26, Lr28, Lr34, Lr35, Lr50. The number of Lr genes in the combinations ranged from two to five (Davoyan, 2022). The reasons for the rare use of the translocation with Lr51 are unclear. It is hypothesized that this translocation may negatively affect for agronomically valuable traits and reduce grain productivity overall (Helguera et al., 2005). However, there is no data on prebreeding studies of the T1BS.1BL-1S#F7L-1BL translocation; information on the impact of this translocation for grain yield and bread making quality is absent. The purpose of our research is: based on the results of a three-year study of spring bread wheat lines-analogous with T1BS.1BL-1S#F7L-1BL translocation, identify the influence of translocation on cytological stability, agronomically important traits, grain productivity and bread making quality, and thus determine its breeding value.

## Materials and methods

The material used included the following samples:

Spring bread wheat cultivar Saratovskaya 68, which belongs to the erythrospermum variety, is valuable for quality, resistant to drought, susceptible to leaf and stem rust pathogens, and contains a gene Lr10 ineffective against the leaf rust pathogen (Gultyaeva et al., 2020).

Spring bread wheat cultivar Saratovskaya 76 – a standard approved by the State Commission of the Russian Federation for the Testing and Protection of Selection Achievements for the Saratov region.

L6 and L7 lines are analogues of spring bread wheat cultivar Saratovskaya 68. Pedigree: Saratovskaya 68/F-7- 3*3// Saratovskaya 68. Lines analogues were obtained after crossing with line F-7-3 and triple backcrossing with cultivar Saratovskaya 68, where the pedigree of the near-isogenic line F-7-3 = Neepawa*6/Triticum speltoides, contains translocation T1BS.1BL-1S#F7L-1BL with the gene Lr51. The line F-7-3 was kindly provided by Doctor of Biological Sciences E.I. Gultyaeva (FSBSI VIZR, Saint Petersburg, Pushkino). Lines L6 and L7 belong to the erythrospermum variety and are highly resistant to the leaf rust pathogen.

Cytogenetic studies. The cytological stability was assessed by studying chromosome behavior during microsporogenesis in meiosis. Meiosis stages in microsporogenesis were studied on temporary squashed preparations of anthers. Spikes of lines L6 and L7 were cut before emerging from the leaf sheath and fixed in a mixture of 96 % ethanol and glacial acetic acid (3:1). Twenty-four hours after fixation, the material was transferred to 70 % ethyl alcohol, where it was stored until analysis at t = +2–4 °C. Schiff reagent was used as a dye staff. For each line, 100–200 microsporocytes at meiosis stages (metaphase I and II, anaphase I and II, telophase I and II, tetrads) were analyzed. Slides were analyzed on a microscope Axio Scope A 1 (Carl Zeiss) with objectives N-ACHROPLAN 40x/0.65 and N-ACHROPLAN 100x/1.25 0;1.

Phytopathological analysis. Under field conditions, the degree of resistance to the leaf rust pathogen was evaluated according to the reaction type of the Mains and Jackson scale (1926), where: 0 – absence of symptoms; 0; – necroses without pustules; 1 – very small pustules surrounded by necrosis; 2 – medium-sized pustules surrounded by necrosis or chlorosis; 3 – medium-sized pustules without necrosis; 4 – large pustules without necrosis, X – pustules on the same leaf of different types, with chloroses and necroses present. Plants with infection types 0–2 were classified as resistant (R), and 3, 4, and X (S) – as susceptible. The percentage of rust infection was assessed according to the R.F. Peterson et al. (1948) scale.

For laboratory evaluation, mixed Saratov populations of Puccinia triticina with artificially increased presence of pathotype pp19, virulent to gene Lr19 were used. Seedlings (three-leaf stage), grown in soil-filled containers, were sprayed with an aqueous spore suspension of the population with the addition of Tween 80 detergent. The suspension concentration was 1 mg inoculum per 1 mL of water. After plant inoculation, a dark period was created for 20 hours at 100 % relative humidity, then seedlings were grown at a temperature of 20–22 °C, with a photoperiod of 16 h day/8 h night. The type of reaction to the pathogen of seedlings was determined according to the Mains and Jackson scale (1926).

Molecular genetic analysis. For PCR analysis, DNA was isolated from five-day-old wheat seedlings using the cetyltrimethylammonium bromide (CTAB) method (Murray, Thompson, 1980). For the identification of the resistance gene Lr51 CAPS-marker was used, for the gene AGA7, located in Ae. speltoides translocation, containing Lr51. Primers S30-13L 5ʹ-GCATCAACAAGATATTCGTTATGACC-3ʹ and AGA7-759R 5ʹ-TGGCTGCTCAGAAAACTGGACC- 3ʹ proposed in the work by M. Helguera et al. (2005) were used. Amplification was carried out on T100 Thermal Cycler (BioRad) amplifiers. Restriction was performed using the endonuclease PstI (SibEnzyme). Separation of amplification products was carried out in a 2 % agarose gel stained with ethidium bromide. The positive control was near-isogenic line F 7-3, and the negative control was the recipient cultivar Saratovskaya 68. GeneRulerTM 100bp DNA Ladder (“Eurogene”) was used as a molecular weight marker. PCR was performed in duplicate.

Evaluation of grain productivity indicators, physical and baking properties of dough and bread in lines L6, L7, and the standard cultivar Saratovskaya 76 was conducted in 2023–2025. Experimental material was randomly sown in plots of 7 m2 in 3-fold repetition on the experimental field of Federal Center of Agriculture Research of the South-East Region in Saratov. Sowing rate was 400 grains per 1 m2. In addition to phenological observations and direct assessment of grain yield, studies of the elements of main spike productivity were conducted: spike length, number of spikelets and grains, grain weight per spike, spike density, grain set per spikelet (as a generalized fertility indicator), which were determined for 15 spikes of the studied lines and the standard cultivar. Bread making quality traits were evaluated by the content of crude gluten, the strength of which was determined using the Gluten Deformation Meter – IDK-3M (“PLAUN”), as well as by the Chopin alveograph (Chopin Technologies) traits with the baking of experimental samples of bread. The protein content in grain from the 2023–2025 harvests was determined using the Foss Infratec TM 1241 grain analyzer (Foss Analytical A/S).

Meteorological characteristics of the growing years according to the Selyaninov hydrothermal coefficient (HTC) (www. agrometeo.online/articles/gtk.htm, accessed January 26, 2026) by months of the vegetation period are given in Table 1. Of the three years of research, the most favorable meteorological conditions were in 2023 and 2025. Epiphytotics of leaf rust were observed in all three years of research, with the greatest pathogen development observed in the 2023 and 2025 growing seasons

**Table 1.**
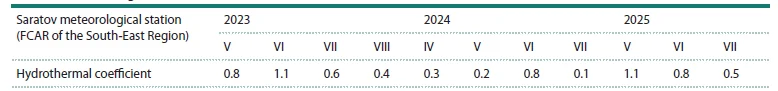
Hydrothermal coefficients for the Saratov meteorological station (Federal Center of Agriculture Research of the South-East Region) for 2023–2025 Note. IV – April, V – May, VI – June, VІІ – July, VIII – August.

The obtained data on lines L6 and L7, recipient and standard cultivars Saratovskaya 68 and Saratovskaya 76 were subjected to a one-way variance analysis with multiple comparisons according to Duncan using the Agros-2.10 breeding and genetic software package.

## Results

**Cytological stability of L6 and L7 lines**

To assess meiotic stability, an integrated indicator called the meiotic index is used. This indicator represents the percentage of normal tetrads relative to the total number of cells studied. Studies have shown that L6 had a meiotic index of 98.2 %, L7 had a meiotic index of 99.0 %, while the recipient cultivar Saratovskaya 68 had a meiotic index of 98.8 %. This indicates that there were no differences between the lines and the recipient cultivar for this indicator. The plants with a meiotic index of 90 % or higher is considered cytologically stable, indicating that both lines are stable. In earlier stages of meiosis, two chromosomes were detected in both lines that were not included in the nuclei in telophase I in an amount of 0.07 %. Among the mononuclear pollen, micropollen was detected in L6 and L7: 0.02 % in L6 and L7, and 0.01 % in Saratovskaya 68.

**Phytopathological analysis of lines L6 and L7 for resistance to leaf rust. Identification of the leaf rust resistance gene Lr51 using DNA markers S30-13L and AGA7-759R**

L6 and L7 showed high resistance to P. triticina in 2023–2025, both during artificial infection and during natural epiphytotics of the pathogen (Table 2). Considering that plants were at the three-leaf stage during artificial infection with P. triticina and field evaluations were conducted during milky or milky-wax ripeness, this suggests a juvenile nature of the resistance gene in the lines L6 and L7. At the same time, the recipient cultivar Saratovskaya 68 and the standard cultivar Saratovskaya 76 were susceptible to the leaf rust pathogen. Results of gene Lr 51 identification by PCR analysis using DNA markers S30- 13L and AGA7-759R in the analyzed lines L6 and L7 showed its presence (see the Figure).

**Table 2.**
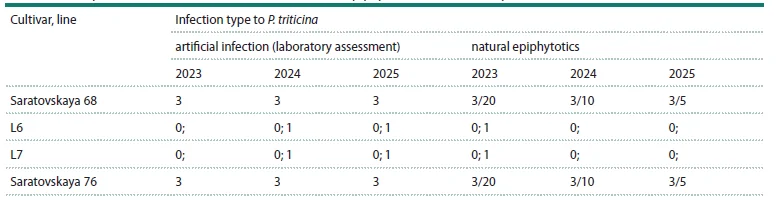
Characteristics of resistance of spring bread wheat lines L6 and L7, cultivars Saratovskaya 68 and Saratovskaya 76 to Puccinia triticina in the field (natural epiphytotics) and laboratory (artificial infection) Note. Laboratory assessment at the 3-leaf stage. The assessment was carried out during natural epiphytotics of P. triticina in the experimental field of FCAR of the South-East Region, Saratov.

**Fig. 1.**
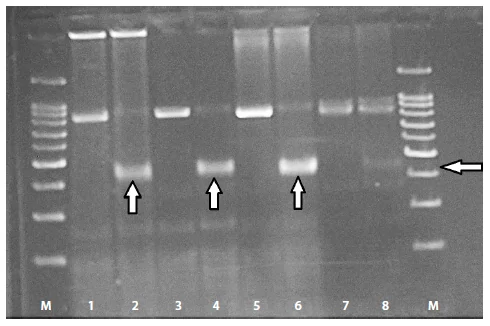
Electrophoregram of amplification and restriction products using primers S30-13L and AGA7-759R, diagnosing the Lr51 gene M – marker of DNA lengths, 100 bp (“Eurogen”). The amplification product with a marker size of 819 bp was determined in line F7-3 Lr51 (1) and in lines-analogous L6 (3), L7 (5). Tracks 2, 4, 6 are restriction products of PstI amplicons, respectively, F7-3 Lr51, L6 and L7. Track 7 is the recipient Saratovskaya 68. Track 8 is the restriction product of the PstI amplicon of the recipient Saratovskaya 68.

**Phenology, grain productivity and bread making quality traits in the lines L6 and L7, recipient cultivar Saratovskaya 68 and standard cultivar Saratovskaya 76**

In Table 3 the traits of the germination–heading period, plant height, and lodging resistance in the 2023–2025 growing seasons are given for lines L6, L7, recipient and standard cultivars. During the 2023–2025 growing seasons, the germination– heading period of the lines L6, L7 and the Saratovskaya 68, Saratovskaya 76 cultivars was almost the same and the differences were insignificant. Thus, the translocation T1BS.1BL-1S#F7L-1BL has almost no effect on the germination– heading period. In terms of plant height in 2023 and 2025, the lines L6 and L7 differed insignificantly from the Saratovskaya 68 and Saratovskaya 76 cultivars, but in 2024, line L6 significantly outperformed both cultivars, and in 2025, the differences were between the lines – L7 exceeded L6. On average, for the period 2023–2025, there were no differences in plant height between cultivars (Saratovskaya 68 – 86 cm, Saratovskaya 76 – 85 cm) and lines (L6 – 88 cm, L7 – 89 cm), as well as between the lines themselves. Thus, the translocation T1BS.1BL-1S#F7L-1BL in the Saratovskaya 68 cultivar did not lead to a significant change in plant height. Plant height affects plant resistance to lodging. In our studies, L7 was significantly inferior in this trait to both cultivars and L6 in the 2023 growing season. In 2024, L7 was inferior to the Saratovskaya 76. In 2025, there were no differences between lines and cultivars. On average, for the period 2023–2025, there were no differences for lodging resistance between cultivars (Saratovskaya 68 – 4.0 and Saratovskaya 76 – 4.0) and lines (L6 – 4.0 and L7 – 3.7), or between lines themselves. However, a decrease in lodging resistance was observed in L7. Comparisons of L6, L7 for grain productivity and its elements in the main spike made it possible to identify the influence of the translocation T1BS.1BL-1S#F7L-1BL on these traits (Tables 4 and 5).

**Table 3.**
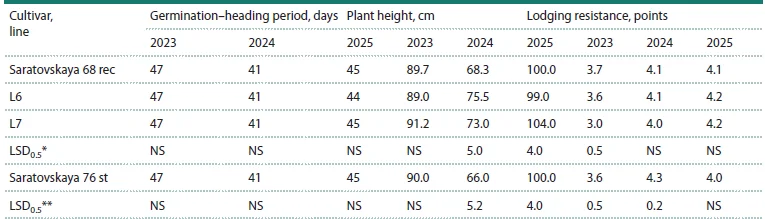
The period of germination–heading, plant height, resistance to lodging in L6, L7 and the cultivars Saratovskaya 68 and Saratovskaya 76 for the growing seasons of 2023–2025 Note. The least significant difference for the 5 % significance level between L6, L7 and: Saratovskaya 68 (*) and Saratovskaya 76 (**). Here and in Tables 4–6: NS – no difference.

**Table 4.**
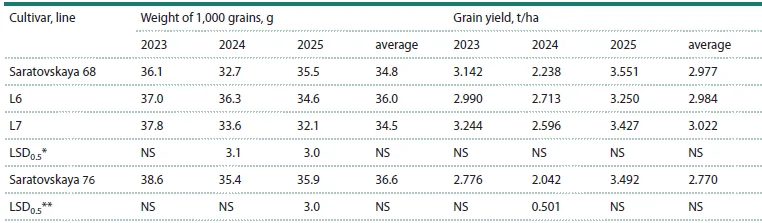
The productivity and weight of 1,000 grains of spring bread wheat lines L6, L7 and cultivars Saratovskaya 68, Saratovskaya 76 for the growing seasons 2023–2025 Note. The least significant difference for the 5 % significance level between L6, L7 and: Saratovskaya 68 (*) and Saratovskaya 76 (**).

**Table 5.**
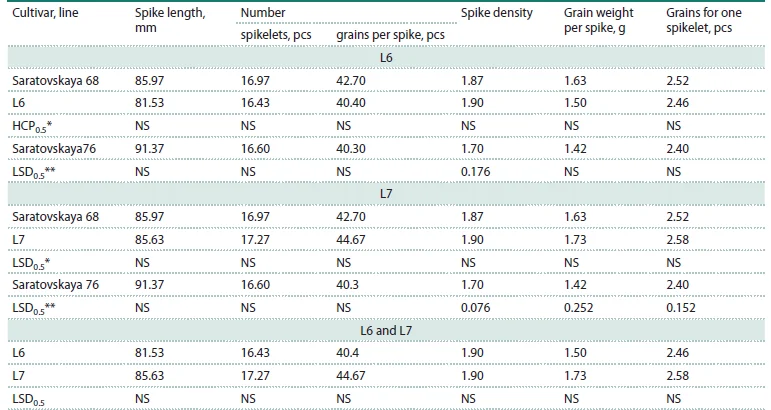
The elements of the main spike productivity of spring bread wheat lines L6, L7, recipient cultivar Saratovskaya 68 and standard cultivar Saratovskaya 76 on average for the growing seasons 2023–2025 Note. The least significant difference for the 5 % significance level between the line and: the recipient cultivar Saratovskaya 68 (*) and the standard cultivar Saratovskaya 76 (**).

It was found that the presence of the translocation T1BS.1BL-1S#F7L-1BL in both lines L6 and L7 during the

study years did not significantly affect for grain yield compared to the Saratovskaya 68 (Table 4). However, differences were observed compared to the standard Saratovskaya 76 during the 2024 growing season; the cultivar was significantly inferior to both lines (Table 4). As shown by the hydrothermal coefficients data, drought from strong to weak was observed during the 2024 growing season (Table 1). Thus, there is reason to assume that L6 and L7 with the translocation T1BS.1BL-1S#F7L-1BL are more drought-resistant than the standard Saratovskaya 76, but are of the same level as the recipient Saratovskaya 68. One of the factors that affects grain productivity is the 1,000 grain weight. On average, there were no significant differences in this trait between lines L6, L7 and cultivars Saratovskaya 68 and Saratovskaya 76 over the period 2023–2025, but there were differences in individual years. L6 surpassed the recipient Saratovskaya 68 for 1,000 grain weight in 2024, and L7 was inferior to both Saratovskaya 68 and Saratovskaya 76 in 2025 (Table 4). Analysis of the elements of the main spike productivity revealed that L6 and L7 with the translocation T1BS.1BL-1S#F7L-1BL did not differ significantly from the recipient Saratovskaya 68 for all parameters. Thus, there is reason to assess the effect of the translocation T1BS. 1BL-1S#F7L-1BL on the elements of the main spike productivity as neutral. However, significant differences for spike density were found between the standard cultivar Saratovskaya 76 and both L6 and L7. Furthermore, L7 significantly exceeded the standard cultivar for grain weight per spike and grains for one spikelet. There were no significant differences between the L6 and L7 lines for terms of the elements of the main spike productivity (Table 5).

The following results were obtained for bread making quality traits in the studied lines. Grain protein content in L6 and L7 did not differ significantly from either the recipient or standard cultivars. Similarly, no significant differences were found for gluten content or strength compared to the recipient cultivar Saratovskaya 68. However, gluten strength in the lines L6 and L7, as measured by the IDK-3M gluten testing device, was stronger than that of the standard cultivar Saratovskaya 76 (Table 6).

**Table 6.**
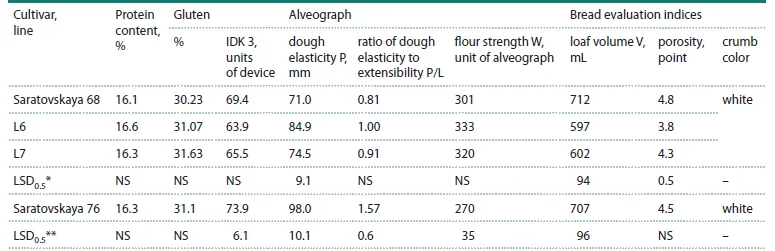
Bread making quality traits for spring bread wheat lines L6, L7, recipient cultivar Saratovskaya 68 and standard cultivar Saratovskaya 76 on average for the growing seasons 2023–2025 Note. The least significant difference for the 5 % significance level between L6, L7 and: the recipient cultivar Saratovskaya 68 (*) and the standard cultivar Saratovskaya 76 (**).

According to the alveograph indices, L6 demonstrated significantly higher dough elasticity (P) compared to the recipient cultivar and the line L7, but L7 did not differ from the Saratovskaya 68 for this parameter. In terms of the elasticityto- extensibility ratio (P/L) and flour strength (W), the lines L6 and L7 did not differ significantly from the recipient cultivar. However, they significantly reduced the loaf volume, and L6 also reduced the bread porosity rating. The comparison of the L6, L7, and the Saratovskaya 76 cultivar for bread making quality traits revealed greater differences. In terms of elasticity, both lines were inferior to the standard cultivar, while L6 did not differ in terms of the elasticity-to-extensibility ratio, but L7 had a lower value. For flour strength trait, both lines significantly exceeded the Saratovskaya 76, but had a significantly lower loaf volume, and there were no differences in crumb porosity. The translocation T1BS.1BL-1S#F7L-1BL in the lines L6 and L7 did not change the color of the bread crumb; both lines, like the recipient and the standard cultivars, had a white crumb (Table 6).

## Discussion

Wild relatives of bread wheat possess many genes of agronomic interest and may be valuable sources of resistance to diseases, insects, and extreme environmental factors. This primarily concerns species closely related to bread wheat, including Ae. speltoides Tausch 2n = 14, genome S. This species belongs to the secondary gene pool of bread wheat (Shneider et al., 2008). Overall, this species is attractive due to its immunity to a number of diseases and its greater capacity for recombination with bread wheat chromosomes. This greater capacity for recombination is due to both the relatives of the S and B genomes and the presence of genes in the S genome that suppress the homologous conjugation gene system (Ph) and allow synapsis of homeologous chromosomes. As noted above, 12 genes transferred from this species were registered in the Catalogue of Wheat Gene Symbols, including six genes for resistance to leaf rust, three to stem rust, and three to powdery mildew (grainsgenes.org/GG3/wgc, accessed February 11, 2026). The 1S, 2S, 3S, 5S, 6S, and 7S chromosomes of Ae. speltoides were involved in the process of recombinogenesis with the chromosomes of bread wheat. The most of the transferred genes for resistance to pathogens from this species included regions of the 2S chromosome (Lr35, Sr32, Sr39, Sr47). From the remaining chromosomes of Ae. speltoides there were one or two transfers with resistance genes. Translocations were transferred from chromosome 1S to chromosome 1B: 1BL.1SS (Pm32) and 1BS.1BL-1S#F7L- 1BL (Lr51). Thus, both arms of chromosome 1S were involved in the exchanges (grainsgenes.org/GG3/wgc, accessed February 11, 2026).

In our studies, based on the lines-analogous of spring bread wheat cultivar Saratovskaya 68 with the translocation 1BS.1BL-1S#F7L-1BL with the Lr51 gene, we studied for cytological stability, determined the effect on morpho-biological and phenological traits, the effectiveness of protection against the leaf rust pathogen, as well as grain productivity and the bread making quality. Unfortunately, we were unable to find reports of similar studies from the sources available to us. Therefore, a comparison of the results of our studies with the results of other studies is not possible.

One factor limiting the practical application of introgression lines is their instability, leading to the rapid loss of alien genetic material. This instability is due to disturbances in the meiotic cycle of hybrid plants, causing the formation of nonfunctional gametes (Orlovskaya et al., 2015). Lines with alien translocations obtained by the method of induced homeologous exchanges or spontaneous exchanges between chromosomes of an alien species and chromosomes of common wheat are characterized by greater stability (Sears, 1972; Friebe et al., 2000). The translocation T1BS.1BL-1S#F7L-1BL used by us with the Lr51 gene in the original F-7-3 line was obtained during crosses (backcrossing) of the cultivar Neepawa with Ae. speltoides source F. That is, there is reason to assume a homeological exchange between of the Ae. speltoides and bread wheat chromosomes. It is known that Ae. speltoides species itself carries both the gene(s) that suppress the action of the Ph genes (paired conjugation) and the gametocidal genes that affect microsporogenesis and pollen viability, but the localization of these genes does not affect chromosome 1S (grainsgenes.org/GG3/wgc, accessed February 11, 2026). In this regard, there was reason to expect minor violations of cytological stability in the lines L6 and L7 compared to the recipient cultivar Saratovskaya 68. In general, our study determined the cytological stability in the lines L6 and L7, the meiotic index reached 98.2 and 99.0 %, and 98.8 % in the recipient cultivar Saratovskaya 68.

The Lr51 gene was highly effective against the Saratov populations of P. trticina in the growing seasons of 2023– 2025, which coincides with the data of E.I. Gultyaeva for the effectiveness of this gene in the Russian Federation (Gultyaeva et al., 2024). The presence of the translocation T1BS.1BL- 1S#F7L-1BL in L6 and L7 did not significantly affect for plant height and lodging resistance either in comparison with the recipient cultivar Saratovskaya 68 or the standard Saratovskaya 76. However, it should be noted that the lodging resistance in L7 decreased in the 2023 and 2024 growing seasons. In the lines-analogous of Saratovskaya 68, the duration of the germination-heading period was at the level of the recipient and the standard cultivars. For grain productivity, the lines with the translocation T1BS.1BL-1S#F7L-1BL did not differ significantly from the recipient cultivar, either in individual years or on average for the period 2023–2025. However, compared with the standard cultivar Saratovskaya 76, the L6 and L7 lines significantly outperformed Saratovskaya 76 in 2024, but there were no differences in 2023 and 2025 and for the period 2023–2025 on average. The detailed analysis of the elements of the main spike productivity on average for 2023–2025 for the lines with the translocation T1BS. 1BL-1S#F7L-1BL showed no differences with the recipient cultivar Saratovskaya 68 for all traits. Nevertheless, compared with the standard cultivar Saratovskaya 76, there were significant differences in favor of the lines with the translocation for spike density, and for L7 also in terms of grain weight per spike and grains for one spikelet. The presence of the translocation T1BS.1BL-1S#F7L-1BL in L6 and L7 did not significantly affect for protein content, nor flour and bread indices, except for loaf volume and porosity. Both lines were inferior to the recipient cultivar. By the comparisons with the standard cultivar Saratovskaya 76, the results were mixed. The lines with the translocation were inferior to Saratovskaya 76 for elasticity, the elasticity-to-extensibility ratio, and loaf volume, but surpassed it for flour strength. Based on the above results, M. Helguera’s hypothesis (Helguera et al., 2005) on the negative impact of translocation with Lr 51 gene, associated with its large size, is not confirmed. However, different interactions of the translocation T1BS.1BL-1S#F7L-1BL with different recipient genotypes are possible, which will change the impact on agronomically valuable traits. The different effects were observed in near-isogenic lines of spring bread wheat carrying translocation 7DL-7Ae#1L·7Ae#1S with the gene Lr29 in the Saratovskaya 68 and Saratovskaya 70 cultivars (Sibikeev et al., 2022).

## Conclusion

Overall, the impact of translocation T1BS.1BL-1S#F7L-1BL in the Saratovskaya 68 genotype is assessed as neutral based on cytological stability and the entire range of breedingvaluable traits. Furthermore, this translocation did not reduce grain productivity under the drought conditions of 2024, indicating that its presence does not reduce drought tolerance. The comparisons with the standard cultivar Saratovskaya 76 demonstrated sufficient breeding significance for lines L6 and L7 with translocation T1BS.1BL-1S#F7L-1BL in the Saratovskaya 68 genotype.

## Conflict of interest

The authors declare no conflict of interest.
